# Rutaecarpine Protects Human Endothelial Cells from Oxidative-Stress-Induced Apoptosis via TRPV1- and AhR-Mediated Nrf2 Activation

**DOI:** 10.3390/antiox14050616

**Published:** 2025-05-21

**Authors:** Chae Yeon Kim, Gi Ho Lee, Seung Yeon Lee, Anh Thi Ngoc Bui, Hye Gwang Jeong

**Affiliations:** College of Pharmacy, Chungnam National University, Daejeon 34134, Republic of Korea; chaeyeon05@o.cnu.ac.kr (C.Y.K.); ghk1900@cnu.ac.kr (G.H.L.); sy9842@o.cnu.ac.kr (S.Y.L.); anhbui@cnu.ac.kr (A.T.N.B.)

**Keywords:** rutaecarpine, endothelial dysfunction, oxidative stress, antioxidant enzymes, apoptosis

## Abstract

Endothelial cells play a crucial role in cardiovascular health by maintaining vascular homeostasis, regulating blood flow and vascular wall permeability, and protecting against external stressors. Oxidative stress, particularly excessive reactive oxygen species (ROS), disrupts cellular homeostasis and contributes to endothelial cell dysfunction. Rutaecarpine (RUT), an indolopyridoquinazolinone alkaloid isolated from *Evodia rutaecarpa*, has cytoprotective potential. However, the molecular mechanism underlying its cytoprotective activity in endothelial cells remains unclear. In this study, we investigated the protective effects of RUT against H_2_O_2_-induced apoptosis in human EA.hy926 endothelial cells and explored its underlying mechanism of action. RUT enhanced nuclear factor erythroid 2-related factor 2 (Nrf2) activation by increasing its expression and phosphorylation, resulting in the upregulation of antioxidant enzymes (GCLC, NQO1, and HO-1). RUT increased the level of the anti-apoptotic marker (Bcl-2) while inhibiting apoptotic markers (cleaved caspase-3 and Bax) in H_2_O_2_-induced apoptotic cells. Mechanistic analysis revealed that RUT activates Nrf2 through two pathways: TRPV1-mediated PKCδ/Akt phosphorylation and aryl hydrocarbon receptor (AhR)-dependent Nrf2 expression. These findings suggest that RUT exerts protective effects against oxidative-stress-induced apoptosis by controlling the Nrf2 signaling pathway in endothelial cells.

## 1. Introduction

Endothelial cells are essential for maintaining vascular homeostasis, primarily through the regulation of blood flow, barrier function, and immune responses. However, oxidative stress and endothelial cell apoptosis are key contributors to the progression of cardiovascular diseases, including atherosclerosis and hypertension. Oxidative stress promotes hypertrophy, apoptosis, and inflammation by activating redox-sensitive signaling pathways and transcription factors [1]. This condition occurs when reactive oxygen species (ROS) levels exceed the body’s antioxidant defenses and the ability to repair oxidative damage is impaired [2]. When oxidative stress overwhelms the cellular protective mechanisms, excessive ROS accumulation disrupts redox homeostasis, ultimately leading to endothelial dysfunction and apoptosis [3,4].

Nuclear factor erythroid 2-related factor 2 (Nrf2) plays a central role in regulating cellular defense mechanisms and works primarily by regulating the expression of genes associated with antioxidant responses, detoxification, and preservation of cellular homeostasis. Nrf2 is a redox-sensitive transcription factor activated under oxidative stress or other cellular stress conditions such as inflammation, UV radiation, and toxins [5,6]. Once activated, Nrf2 translocates to the nucleus, where it binds to antioxidant response elements (AREs) in the promoter regions of target genes, initiating the transcription of protective genes such as oxygenase-1 (HO-1) and NAD(P)H quinone dehydrogenase 1 (NQO1) [7,8]. Nrf2 activity is highly regulated at various levels, including through protein interactions, post-translational modifications, and cellular stress responses. The primary mechanism of Nrf2 regulation involves its interaction with Kelch-like ECH-associated protein 1 (Keap1). Under basal conditions, Nrf2 is primarily retained in the cytosol through its interaction with Kelch domain of Keap1, which functions as an adapter molecule for CUL-E3 ligase, promoting ubiquitination and degradation of Nrf2. In response to oxidative stress, Keap1 dissociates from CUL-E3 ligase and Nrf2 translocates into nucleus, triggering the transcription of antioxidant genes [9,10]. Besides the Keap1-Nrf2 pathway, the activity of Nrf2 is regulated through post-translational modifications. Phosphorylation of Nrf2 by protein kinase C (PKC) and AMP-activated protein kinase (Akt) stabilizes or enhances Nrf2 activity [11,12]. For example, phosphorylation of Nrf2 by GSK-3β and PKC prevents its interaction with Keap1, leading to its translocation to the nucleus [13,14]. The aryl hydrocarbon receptor (AhR) is another regulator of Nrf2. AhR directly enhances Nrf2 transcription by binding to xenobiotic response elements (XREs) on the Nrf2 promoter, leading to increased levels of Nrf2 protein [15]. In contrast, AhR activation can cause a change in Keap1/Nrf2 levels, thereby reducing the ability of AhR to degrade Nrf2. This allows Nrf2 to accumulate in the nucleus and enhance antioxidant responses [16].

Recently, natural compounds with Nrf2-activating properties have gained attention because of their potential to protect endothelial cells from oxidative stress. Several phytochemicals have been shown to modulate Nrf2 signaling through different upstream regulators, leading to enhanced cellular defense mechanisms. Rutaecarpine (RUT), a bioactive alkaloid extracted from *Evodia rutaecarpa*, exerts multiple pharmacological effects, including anti-inflammatory, antioxidant, and cardiovascular protective effects. In our previous studies, we demonstrated that RUT induces nitric oxide synthesis by promoting eNOS phosphorylation, mediated by TRPV1-dependent CaMKII and CamMKKβ/AMPK signaling in human endothelial cells [17]. RUT also induces CYP1A1 activation in an AhR- and calcium-dependent manner [18]. Moreover, we report that RUT defends against hepatotoxicity by enhancing antioxidant enzymes levels through the CaMKII-Akt and Nrf2/ARE pathways [19]. However, the role of RUT in regulating Nrf2 activity and apoptosis in endothelial cells has not been sufficiently explored.

In this study, we investigated the potential of RUT in protecting endothelial cells from apoptosis and the underlying mechanisms of this protection. Using human endothelial cells (EA.hy926), we investigated the effects of RUT on apoptosis and specifically explored its influence on the Nrf2 signaling pathway in the context of oxidative stress.

## 2. Materials and Methods

### 2.1. Chemicals and Reagents

Rutaecarpine was purchased from Sigma-Aldrich. Dulbecco’s modified Eagle’s medium (DMEM), fetal bovine serum (FBS), penicillin–streptomycin, and trypsin were purchased from Welgene (Gyeongsan, Republic of Korea). Rottlerin, ML385, and CH223191 were purchased from Sigma-Aldrich (St. Louis, MO, USA). SB366791 was obtained from Tocris (Cookson, Bristol, UK). EDTA and BAPTA were acquired from GenDEPOT (Barker, TX, USA). p-PKCδ, p-Akt, and cleaved caspase-3 antibodies were obtained from Cell Signaling Technology (Beverly, MA, USA). NOQ1, Nrf2, Bax, Bcl-2, Akt, PKCδ, and β-actin antibodies were purchased from Santa Cruz Biotechnology (Santa Cruz, CA, USA). p-Nrf2, HO-1, and Lamin B1 were ordered from Bioss Antibodies, Inc. (Woburn, MA, USA). Tetrazole 3-(4,5-dimethylthiazol-2-yl)-2,5-diphenyltetrazolium bromide (MTT) was obtained from the USB Corporation (Cleveland, OH, USA). The Cytotoxicity Assay Kit was sourced from Roche Applied Science (Indianapolis, IN, USA). All the other chemicals used were of the highest commercially available grade.

### 2.2. Cell Culture and Cell Viability Assessment

EA.hy 926 human endothelial cells, obtained from the American Type Culture Collection (Bethesda, MD, USA), were grown in DMEM containing 10% FBS, 100 U/mL penicillin, and 100 μg/mL streptomycin in a 37 °C incubator with 5% CO_2_. Cells were treated with 300 µM H_2_O_2_ to induce oxidative stress. This concentration was selected based on previous studies demonstrating its effectiveness in reducing cell viability and triggering apoptosis in EA.hy 926 cells without causing immediate necrosis [20,21,22]. To assess cell viability, the MTT and LHD assays were performed as reported [17].

### 2.3. RNA Extraction and Real-Time PCR

Total RNA extraction was performed using the RNA Iso Plus Total RNA Extraction Reagent (TaKaRa, Shiga, Japan), and cDNA was synthesized using the BioFact RT Series Kit (BioFact, Daejeon, Republic of Korea). To measure expression of apoptotic genes and antioxidant enzymes, quantitative reverse transcriptase polymerase chain reaction (qRT-PCR) was performed using Bio-Rad CFX Connect Real-Time PCR software, version 1.4.1 (Bio-Rad Laboratories, Hercules, CA, USA). The following primers were used: Nrf2 (NM_010902.5) forward, 5′-CACATCCAGTCAGAAACCAGTGG-3′, Nrf2 (NM_010902.5) reverse, 5′-GGAATGTCTGCGCCAAAAGCTG-3′, HO-1 (M23041.1) forward, 5′-CCAGGCAGAGAATGCTGAGTTC-3′, HO-1 (M23041.1) reverse, 5′-AAGACTGGGCTCTCCTTGTTGC-3′, NQO1 (NM_001286137.2) forward, 5′-CCTGCCATTCTGAAAGGCTGGT-3′, NQO1 (NM_001286137.2) reverse, 5′-GTGGTGATGGAAAGCACTGCCT-3′, Bax (NM_001291430.2) forward, 5′-TCAGGATGCGTCCACCAAGAAG-3′, Bax (NM_001291430.2) reverse, 5′-TGTGTCCACGGCGGCAATCATC-3′, Bcl-2 (NM_001438935.1) forward, 5′-ATCGCCCTGTGGATGACTGAGT-3′, Bcl-2 (NM_001438935.1) reverse, 5′-GCCAGGAGAAATCAAACAGAGGC-3′, GAPDH (NM_001357943.2) Forward, 5′-GAAGGTGAAGGTCGGAGTCAA-3′; and GAPDH (NM_001357943.2) Reverse, 5′-CTTCCCGTTCTCAGCCATGTA-3′. Expression levels were normalized to that of GAPDH.

### 2.4. Western Blotting

Sample preparation and western blotting were performed as previously described [17]. In brief, cells were lysed and SDS-PAGE performed to separate the proteins. After blocking and 2 rounds of antibody incubation, the membrane blots were developed using Hisol ECL Plus Detection Kit (BioFact, Daejeon, Republic of Korea).

### 2.5. ROS Assay

ROS production in EA.hy 926 cells was analyzed using the redox-sensitive fluorescent dye H_2_DCF-DA. Following treatment with RUT and H_2_O_2_, the medium in each well of a 48-well plate was discarded and the cells were probed with 2 μM H_2_DCF-DA at 37 °C for 30 min and rinsed twice with phosphate-buffered saline (PBS). Fluorescence intensity, as an indicator of ROS levels, was measured using a BioTek Synergy HT microplate reader (BioTek Instruments, Winooski, VT, USA; excitation, 490 nm; emission, 530 nm).

### 2.6. Immunofluorescence

EA.hy926 cells were grown in 48-well plates to 60–80% confluence. Cells were treated with 1–5 µM RUT for 4 h and fixed in 4% paraformaldehyde for 20 min at room temperature, following being blocked with 5% serum for 30 min. After washing with PBS plus 0.2% Tween, the cells were immersed in 0.2% Triton X-100 for 10 min. After that, primary antibodies against Nrf2 and AhR (1:200) were added and the mixture incubated at 4 °C overnight, followed by incubation with Alexa Fluor 532-conjugated secondary antibodies (1:500; Molecular Probes, Eugene, OR, USA) for 2 h at room temperature. 4′,6-Diamidino-2-phenylindole (DAPI; 1:1000 dilution) was used for staining nuclei.

### 2.7. Annexin V and Dead Cell Assay

Apoptosis was examined using the Muse Annexin V and Dead Cell Kit (Merck-Millipore, Burlington, MA, USA) following the manufacturer’s instructions. Briefly, after overnight culture of cells in 48-well tissue culture plate, 300 µL of medium containing the test sample was added to each well. After 24 h of treatment, cells were harvested using trypsin-EDTA and centrifuged. The pellet was removed, PBS was added, and the cells were counted (1 × 10^5^ cells/1 mL/well). Subsequently, the sample (100 µL) was combined with the same volume of Muse Annexin-V and Dead Cell Reagent for 20 min in the dark, and annexin-V/7-AAD-positive cells were quantified using a Muse^®^ Cell Analyzer. Early apoptotic cells were identified by annexin V staining alone, whereas late apoptotic cells were characterized by double staining with annexin V and 7-AAD.

### 2.8. Statistical Analysis

Data are expressed as means ± SD from at least three independent experiments. Statistical analyses were performed using one-way analysis of variance (ANOVA). The Newman–Keuls test was used to compare multiple groups. Statistical significance was determined at a threshold of *p* < 0.01.

## 3. Results

### 3.1. RUT Exerts a Protective Effect on Endothelial Cells Against H_2_O_2_-Induced Oxidative Stress

**Figure 1:**
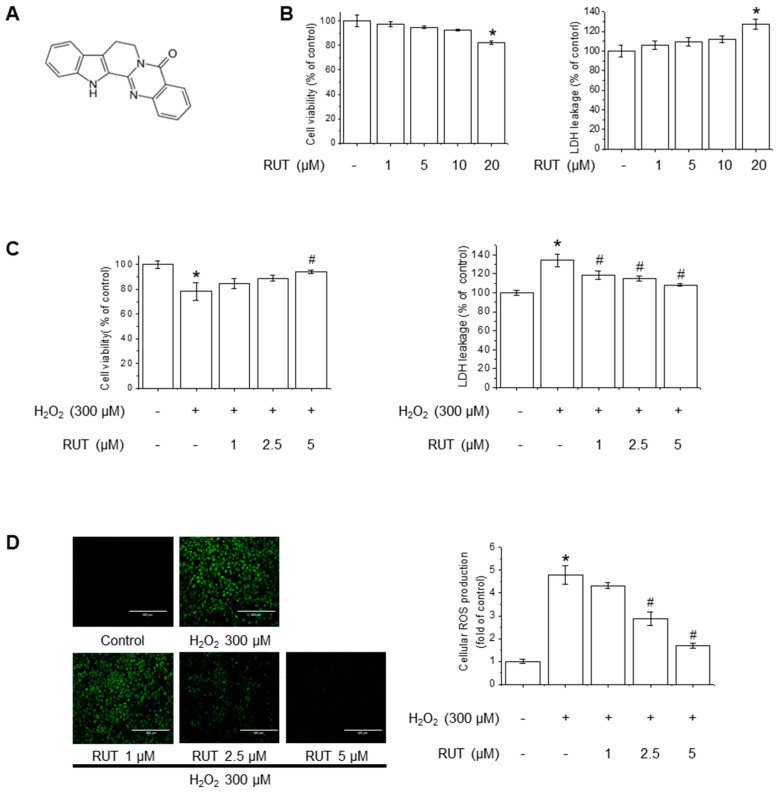
RUT attenuated H_2_O_2_-induced oxidative stress in endothelial cells. (**A**) Chemical structure of RUT. (**B**) Effect of RUT on cell viability and cytotoxicity. Cells were treated with 1–20 µM of RUT for 24 h. Cell viability (**left**) and cytotoxicity (**right**) were assessed by MTT and LDH assays. Effect of RUT on H_2_O_2_-induced cytotoxicity (**C**) and ROS production (**D**). Cells were pretreated with 1–5 µM RUT for 6 h and 300 µM of H_2_O_2_ for 24 h. Cell viability and cytotoxicity were measured by MTT assay and ROS level was measured by H_2_DCF-DA (scale bar, 400 µm; magnification, 20×). Data are expressed as means ± SD (*n* = 3). * Significantly different from the control group at *p* < 0.01. # Significantly different from the H_2_O_2_ treatment group at *p* < 0.01.

The chemical structure of RUT is shown in Figure 1A. The cytotoxicity potential of RUT in endothelial cells was examined using MTT and LDH assays. As shown in Figure 1B, treatment with 20 µM of RUT resulted in significant cytotoxicity, whereas concentrations below 10 µM had no detectable effect on cell viability. Based on these findings, subsequent experiments were conducted using RUT at a maximum concentration of 5 µM. To examine the protective effects of RUT against H_2_O_2_-induced cytotoxicity, we estimated the cell viability and LDH leakage in the presence of H_2_O_2_, an oxidative stress inducer. RUT treatment significantly increased cell survival in a dose-dependent manner following H_2_O_2_ exposure (Figure 1C). As H_2_O_2_ is a well-known generator of oxidative stress that leads to increased ROS production, we further evaluated the effect of RUT on ROS production using the H_2_DCF-DA assay. Notably, cells treated with RUT exhibited a marked reduction in intracellular ROS levels (Figure 1D). These data suggest that RUT exhibits a protective effect against H_2_O_2_-induced cytotoxicity and suppresses ROS generation in cells under oxidative stress.

### 3.2. RUT Ameliorates H_2_O_2_-Induced Apoptosis by Regulating Apoptotic Pathways

**Figure 2:**
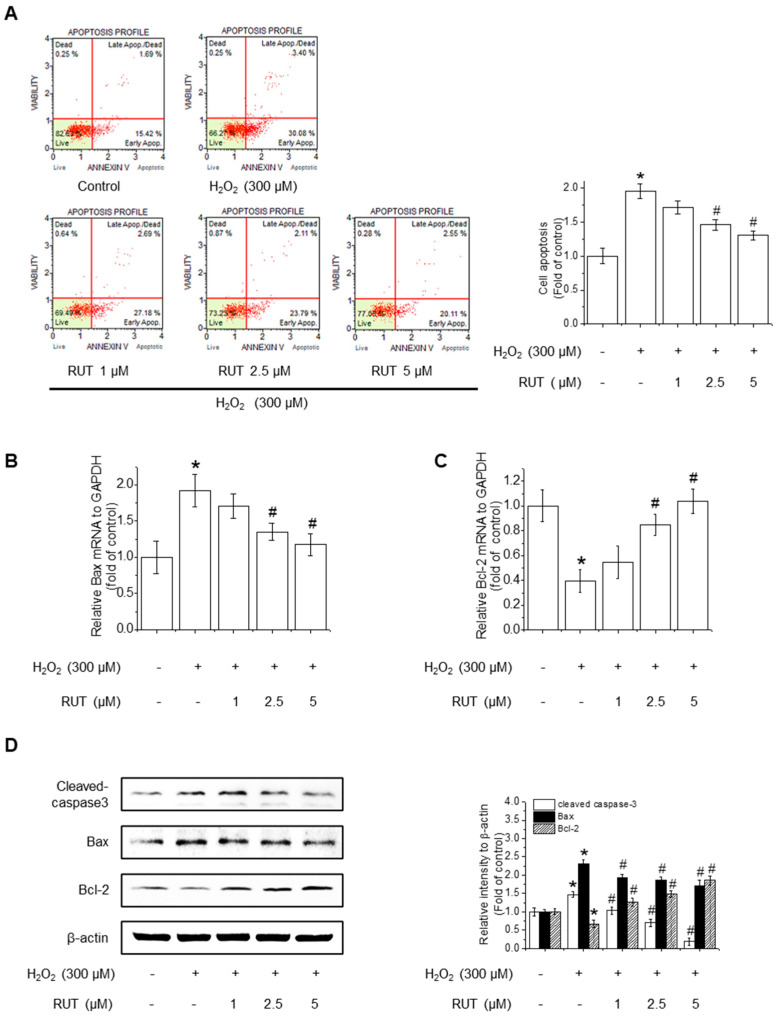
Effect of RUT on H_2_O_2_-induced apoptosis. Cells were pretreated with 1–5 μM RUT for 6 h, followed by 300 μM H_2_O_2_ for 24 h. (**A**) Apoptosis was analyzed using a Muse^®^ Cell Analyzer. The red lines indicate quadrant gates used to distinguish cell populations: lower left (Annexin V^−^/PI^−^_,_ live cells), lower right (Annexin V^+^/PI^−^, early apoptotic cells), upper right (Annexin V^+^/PI^+^, late apoptotic or dead cells), and upper left (Annexin V^−^/PI^+^, Dead cells). Red dots represent individual cells, and percentages reflect the proportion of cells in each quadrant. Bax (**B**) and Bcl-2 (**C**) mRNA levels were analyzed by real-time PCR. (**D**) Protein levels of Bax, Bcl-2, and cleaved caspase-3 were estimated by western blotting. Data are expressed as means ± SD (*n* = 3). * Significantly different from the control group at *p* < 0.01. # Significantly different from the H_2_O_2_ treatment group at *p* < 0.01.

Since excessive ROS accumulation is closely linked to apoptotic cell death, we next examined whether RUT could mitigate H_2_O_2_-induced apoptosis by performing Annexin V staining, followed by cytometry analysis. As shown in Figure 2A, untreated control cells remained primarily in the Annexin V^−^/PI^−^ quadrant, indicating a viable population. Following H_2_O_2_ treatment, there was a remarkable increase in the number of Annexin V^−^/PI^−^ cells, representing early apoptotic cells, along with a rise in Annexin V^+^/PI^+^ cells, indicating late apoptosis or secondary necrosis. Interestingly, treatment with RUT reduced the proportion of Annexin V^+^ cells in a dose-dependent manner, suggesting that RUT effectively inhibited H_2_O_2_-induced apoptosis. Quantification of apoptotic cell populations confirmed a significant reduction in apoptosis in the presence of RUT compared to H_2_O_2_ treatment alone. To determine whether the observed reduction in apoptosis is associated with changes in the intrinsic or extrinsic apoptotic pathways, we assessed the expression of genes involved in mitochondrial apoptosis (Bax and Bcl-2) and caspase activation. qPCR revealed that RUT treatment significantly downregulated the expression of Bax, a proapoptotic protein, and upregulated the expression of Bcl-2, an anti-apoptotic protein (Figure 2B,C). Consistently, RUT treatment decreased Bax protein levels and increased Bcl-2 protein levels in a concentration-dependent manner (Figure 2D). Moreover, RUT treatment reduced the level of cleaved caspase-3, a hallmark of apoptosis. Thus, RUT effectively mitigated H_2_O_2_-induced apoptosis by modulating key apoptotic proteins belonging to the intrinsic apoptotic pathways.

### 3.3. RUT Enhances Antioxidant Activity Through Nrf2 Activation via the PI3K/Akt and PKCδ Signaling Pathways

**Figure 3:**
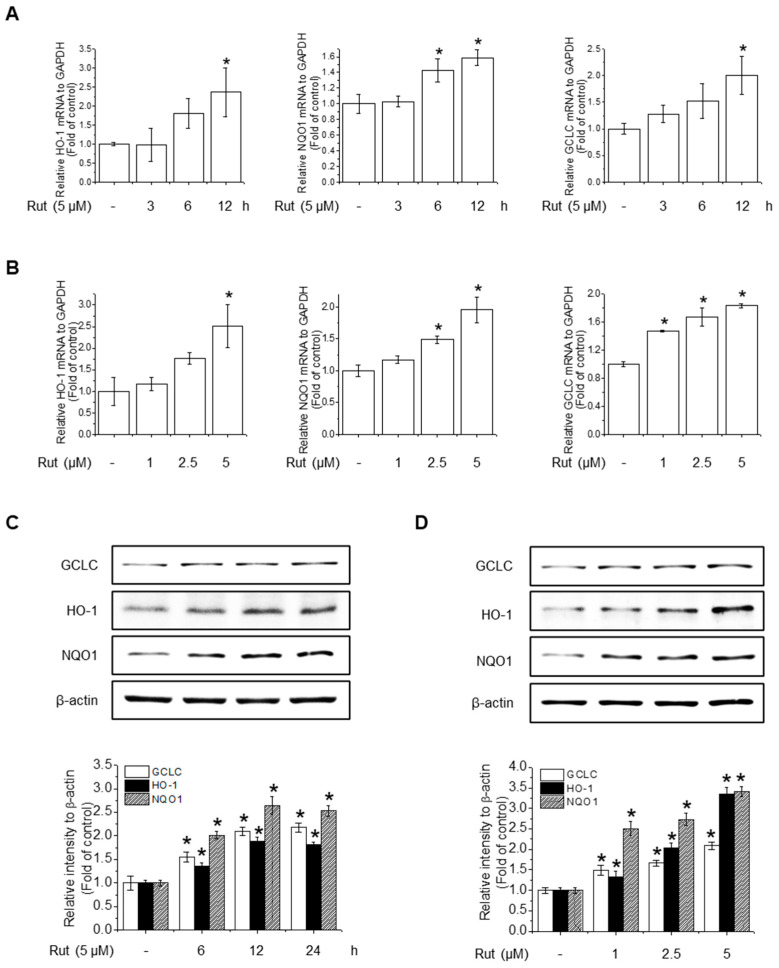
RUT enhances antioxidant enzyme expression in response to oxidative stress. Cells were treated with 5 μM of RUT for 3–12 h (**A**) or with increasing concentrations of RUT (1–5 μM) for 12 h (**B**). mRNA expressions of HO-1, NQO1, and GCLC were quantified by real-time PCR. (**C**,**D**). The effect of RUT on antioxidant enzyme levels was determined by western blotting in time- and dose-dependent manners, respectively. Data are expressed as means ± SD (*n* = 3). * Significantly different from the control group at *p* < 0.01.

To assess the protective effects of RUT on antioxidant activity, we investigated its effect on the gene expression of key antioxidant enzymes. RUT treatment notably increased the mRNA levels of HO-1, NQO1, and GCLC in a time- and concentration-dependent manner (Figure 3A,B). Western blotting confirmed these findings, showing elevated protein levels of the corresponding antioxidant enzymes (Figure 3C,D). These results suggest that RUT enhances the antioxidant defense by upregulating key antioxidant enzymes at both the transcriptional and translational levels.

**Figure 4:**
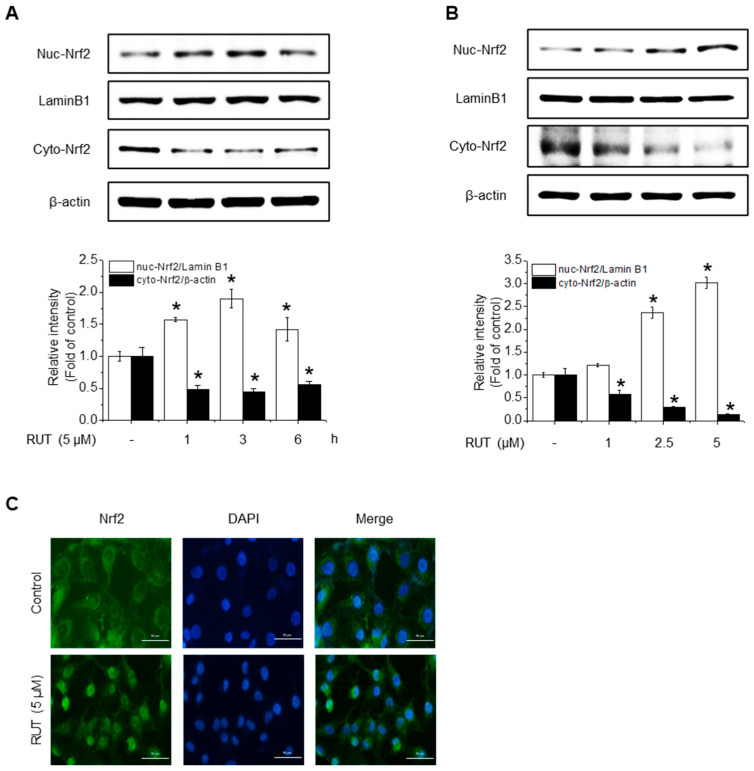

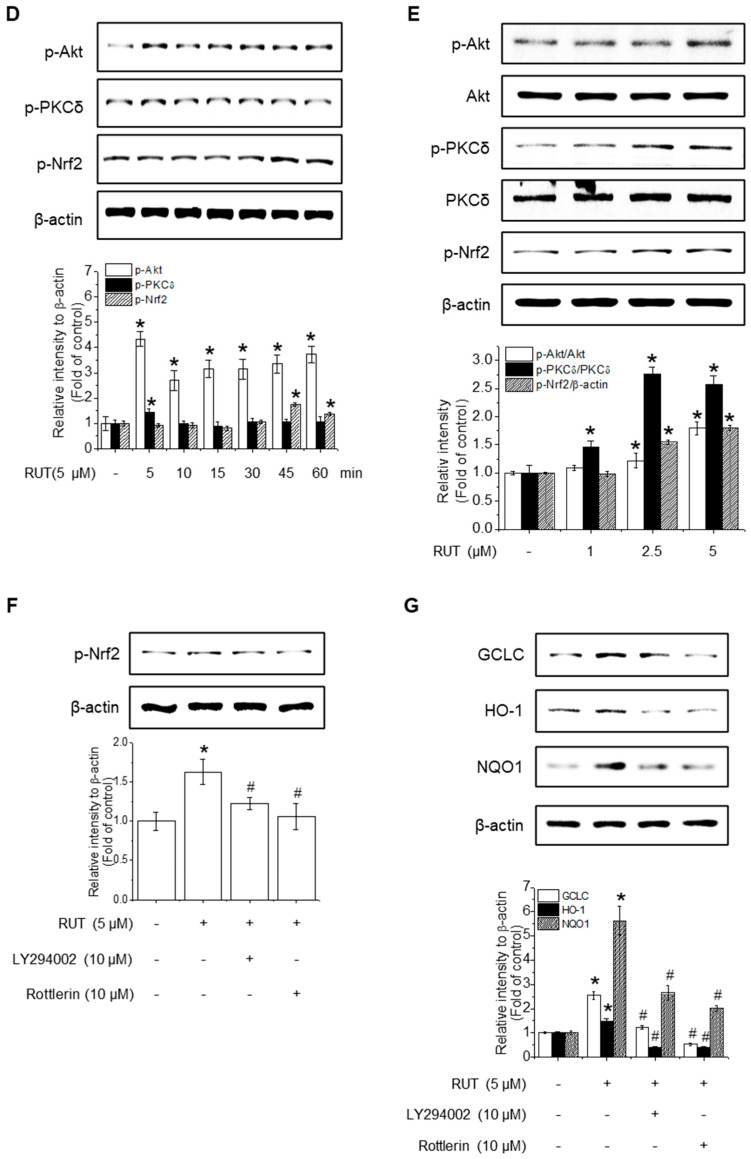
RUT affects Nrf2 regulation by controlling the Akt/PKCδ signaling pathway. Nuclear and cytosolic Nrf2 protein levels were analyzed using western blotting. Cells were treated with 5 μM of RUT for 1–6 h (**A**) or with increasing concentrations of RUT (1–5 μM) for 4 h (**B**). (**C**) Immunofluorescence image of Nrf2 localization upon 5 μM RUT treatment for 4 h (scale bar, 50 µm; magnification, 20×). (**D**,**E**) Effect of RUT on Akt, PKCδ, and Nrf2 phosphorylation in cells treated with 5 μM of RUT for 5–60 min or various concentrations of RUT (1–5 μM). The levels of Nrf2 phosphorylation (**F**) and antioxidant enzymes (**G**) were determined by western blot in the presence of Akt (10 μM LY294002) and PCKδ inhibitors (10 μM Rottlerin) for 1 h and then 5 μM of RUT for 24 h. Data are expressed as means ± SD (*n* = 3). * Significantly different from the control group at *p* < 0.01. # Significantly different from the RUT treatment group at *p* < 0.01.

Given that HO-1, NQO1, and GCLC are established Nrf2 target genes, we next investigated whether RUT exerts its protective effects on Nrf2 activation. To assess this, we examined Nrf2 localization, as its activation promotes its nuclear accumulation. As shown in Figure 4A,B, RUT treatment increased the nuclear translocation of Nrf2 and decreased its cytoplasmic abundance. Immunofluorescence assays confirmed that intranuclear levels of Nrf2 were enhanced following RUT treatment (Figure 4C). We then explored the regulation of Nrf2 by monitoring upstream signaling pathways. Cells treated with RUT exhibited an increase in the phosphorylation levels of Akt, PKCδ, and Nrf2 (Figure 4D,E), suggesting their role in Nrf2 activation. Specifically, Akt showed a marked increase as early as 5 min post-treatment, while the phosphorylation of PKCδ exhibited a modest rise thereafter. Phosphorylation of Nrf2 was observed at 45 min following RUT exposure, suggesting that Nrf2 activation occurs downstream of the Akt/PKCδ signaling pathway (Figure 4E). To confirm their roles, we used the corresponding specific kinase inhibitors (LY294002 for Akt and Rottlerin for PCKδ), which remarkably reduced the phosphorylation of Nrf2 (Figure 4F) and antioxidant enzyme expression (Figure 4G). These findings indicate that RUT activates the Nrf2 signaling pathway through the PI3K/Akt and PKCδ pathways, leading to the enhanced expression of antioxidant enzymes.

### 3.4. TRPV1-Mediated Calcium Influx Regulates Akt/PKCδ Signaling to Promote Nrf2 Activation

**Figure 5:**
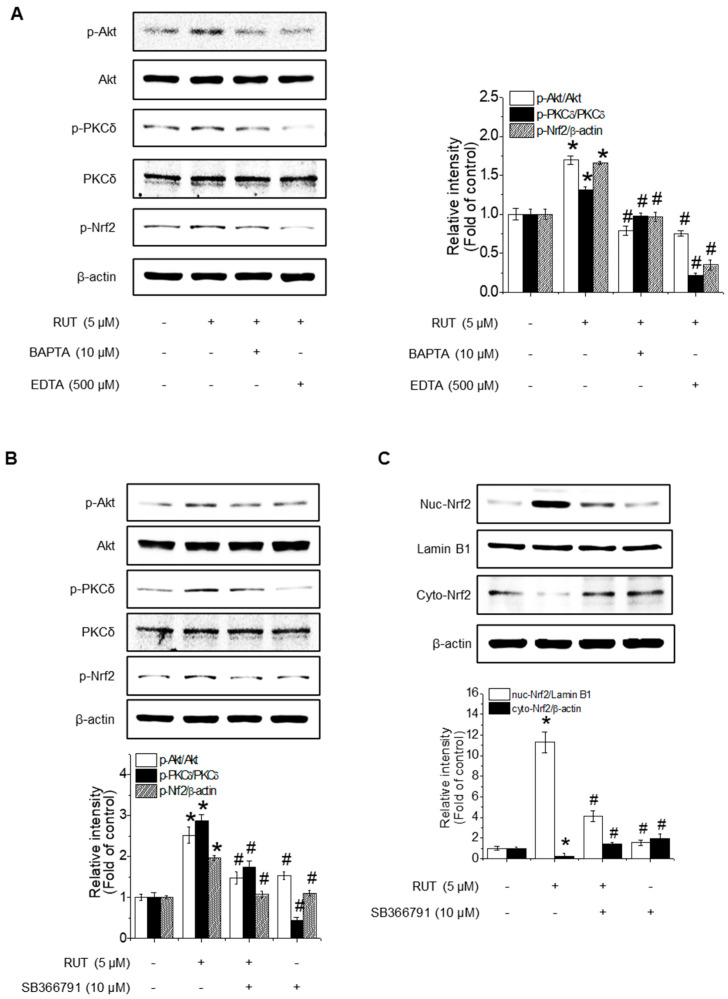
Calcium influx via TRPV1 channels is required for RUT-enhanced Nrf2 phosphorylation through the Akt/PKCδ signaling pathway. The phosphorylation levels of Akt, PKCδ, and Nrf2 were measured by western blotting. (**A**) Cells were pretreated with 10 μM BAPTA and 500 μM EDTA for 1 h, followed by 5 μM RUT. (**B**) Cells were treated with TRPV1-specific inhibitor SB366791 (10 μM) before RUT treatment (5 μM). (**C**) Nrf2 translocation detected by western blotting in the presence of 10 μM SB366791 and 5 μM of RUT for 4 h. Data are expressed as means ± SD (*n* = 3). * Significantly different from the control group at *p* < 0.01. # Significantly different from the RUT treatment group at *p* < 0.01.

As PKCδ activation is known to be calcium-dependent, we investigated whether Ca^2+^ signaling contributes to RUT-mediated Nrf2 activation. Cells pretreated with calcium chelators (EDTA and BAPTA) showed a marked reduction in RUT-induced phosphorylation of Akt, PKCδ, and Nrf2 (Figure 5A), suggesting a role for calcium in Akt/PKCδ signaling. TRPV1 is a well-known calcium-permeable channel and plays a role in regulating PKCδ and PI3K/Akt signaling [23]. To determine whether TRPV1 is functionally involved in the RUT-activated pathway, we used TRPV1-specific inhibitor SB366791. As shown in Figure 5B, inhibition of TRPV1 blocked the phosphorylation of Akt, PKCδ, and Nrf2, indicating TRPV1 plays a critical role in this signaling cascade. Notably, SB366791 treatment disrupted RUT-induced Nrf2 nuclear translocation (Figure 5C), further corroborating the role of TRPV1-mediated calcium influx in Nrf2 activation.

### 3.5. RUT Induces Nrf2 Expression by Activating the AhR Signaling Pathway

**Figure 6:**
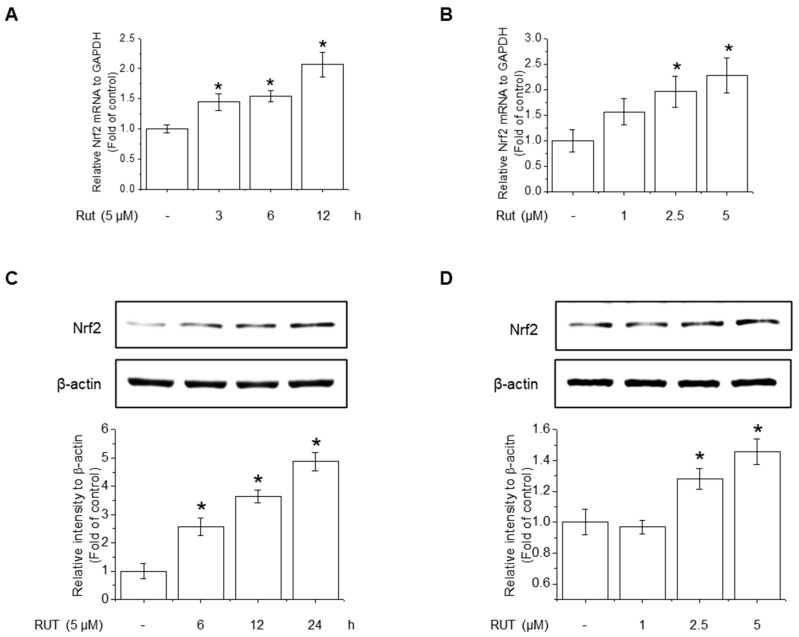
RUT induced Nrf2 mRNA and protein expression. The effect of RUT on Nrf2 mRNA levels was estimated using real-time PCR. (**A**) Cells were treated with 5 μM RUT for 3–12 h and (**B**) 1–5 μM RUT for 12 h. The protein level of Nrf2 was assessed by western blot in cells treated with 5 μM RUT for 24 h (**C**) and RUT (1–5 μM) (**D**) for 24 h. Data are expressed as means ± SD (*n* = 3). * Significantly different from the control group at *p* < 0.01.

**Figure 7:**
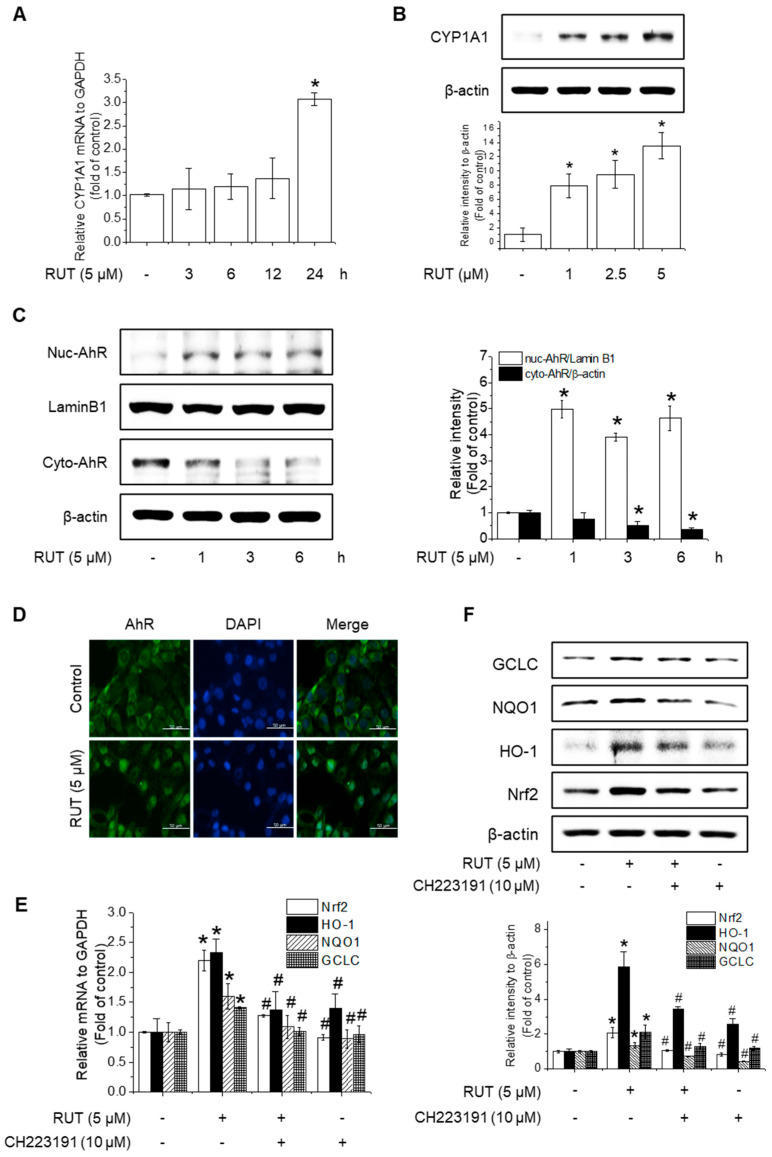
RUT activates the AhR signaling pathway to induce Nrf2 expression. Effects of RUT on CYP1A1 expression. Cells were treated with 5 μM RUT for 3–24 h (**A**) or 1–5 μM RUT for 24 h (**B**). The mRNA and protein levels of CYP1A1 were determined using real-time PCR and western blotting, respectively. AhR translocation in endothelial cells was analyzed by western blotting (**C**) and immunofluorescence (**D**) after treatment with 5 μM RUT (scale bar, 50 µm; magnification, 20×). The expression of Nrf2 and antioxidant enzymes HO-1, NQO1, and GCLC was determined by real-time PCR (**E**) after exposure to 10 μM CH223191 and 12 h treatment with RUT and western blot (**F**) in the presence of 10 μM CH223191 for 1 h, followed by 5 μM RUT for 24 h. Data are expressed as means ± SD (*n* = 3). * Significantly different from the control group at *p* < 0.01. # Significantly different from the RUT treatment group at *p* < 0.01.

The protective effects of RUT manifested through not only enhanced Nrf2 phosphorylation but also likely involved increased Nrf2 expression. RUT treatment increased the mRNA and protein levels of Nrf2 (Figure 6A–D). Therefore, we examined the effect of RUT on the AhR signaling pathway, which controls Nrf2 expression, by monitoring the expression of the AhR target protein CYP1A1. RUT treatment increased CYP1A1 mRNA and protein levels in a time- and concentration-dependent manner (Figure 7A,B). Furthermore, RUT treatment promoted the nuclear translocation of AhR, as determined by western blot and immunofluorescence analyses (Figure 7C,D). Furthermore, pretreatment with the AhR inhibitor CH223191 significantly blocked Nrf2- and Nrf2-mediated enzyme expression (Figure 7E,F). These findings indicate that RUT regulates the expression of Nrf2 and Nrf2-mediated antioxidant enzymes by controlling the AhR signaling pathway.

### 3.6. Nrf2 and AhR Inhibition Counteracts RUT-Mediated Protection Against Oxidative-Stress-Induced Apoptosis

**Figure 8:**
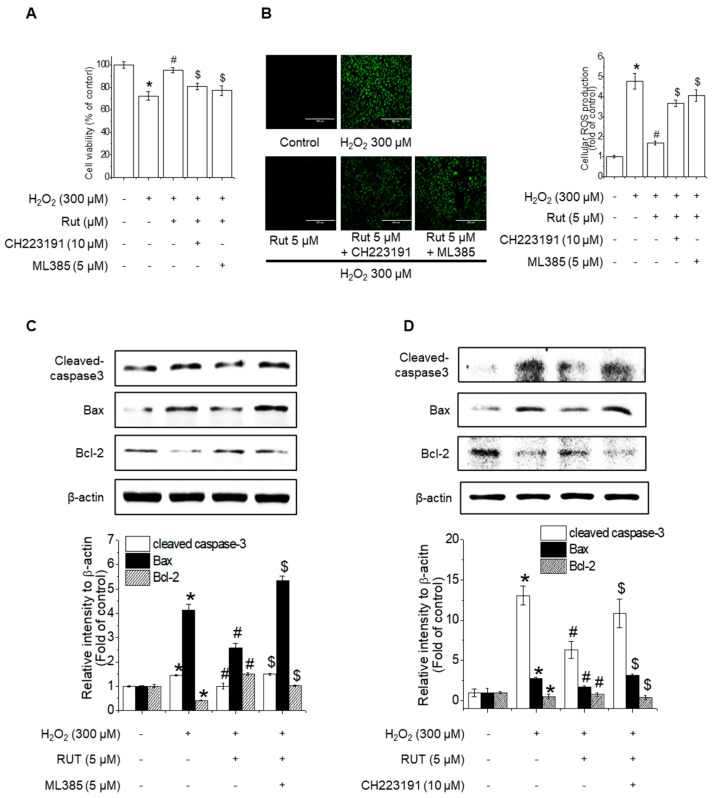
Nrf2 and AhR are critical for the protective effect of RUT against H_2_O_2_-induced apoptosis. (**A**) RUT induced Nrf2 through regulating the AhR/Nrf2 signaling pathway, which had an impact on H_2_O_2_-induced cytotoxicity in endothelial cells. Cells were pretreated with 10 μM CH223191 and 5 μM ML385 for 1 h, followed by 5 μM RUT for 6 h and 300 μM H_2_O_2_ for 24 h. Cytotoxicity was measured using the MTT assay. (**B**) ROS was measured by ROS assay (scale bar, 400 µm; magnification, 20×). (**C**,**D**) The protein levels of Bax, Bcl-2, and cleaved caspase-3 were determined by western blotting. * Significantly different from the control group at *p* < 0.01. # Significantly different from the H_2_O_2_ treatment group at *p* < 0.01. $ Significantly different from the RUT treatment group at *p* < 0.01.

To evaluate the effects of RUT-related Nrf2 and AhR signaling pathway changes on H₂O₂-induced apoptosis in endothelial cells, cell viability assays were performed in the presence of Nrf2 and AhR inhibitors (ML385 and CH223191, respectively). RUT treatment mitigated severe growth defects in cells, whereas pretreatment with these inhibitors counteracted its protective effects (Figure 8A). Similarly, inhibition of Nrf2 and AhR reversed the RUT-mediated reduction in ROS production (Figure 8B). Under oxidative stress conditions, the inhibition of Nrf2 and AhR resulted in increased apoptosis, as evidenced by the upregulation of Bax and cleaved caspase-3 and the downregulation of Bcl-2, effects opposite to those of RUT treatment alone (Figure 8C,D). These findings indicate that Nrf2 and AhR are essential for the protective effect of RUT against H_2_O_2_-induced apoptosis.

## 4. Discussion

ROS damage is one of the main causes of endothelial cell integrity, as it alters cell signaling pathways. ROS impair the ability of the endothelium to regulate vascular tone, blood flow, and inflammation, resulting in the development of atherosclerosis and other cardiovascular conditions [24]. ROS influence various cellular signaling pathways and transcription factors, including PI3K/Akt, MAPK, Nrf2/Keap1, nuclear factor-κB (NF-κB), and the tumor suppressor p53. These pathways are crucial for regulating cell fate and triggering cell death processes such as autophagy and apoptosis [16,25,26]. Given the critical role of ROS-induced apoptosis in endothelial dysfunction and cardiovascular disease, targeting oxidative stress is a potential treatment approach. In this study, we revealed the protective effects of RUT against the oxidative-stress-induced apoptosis of endothelial cells. RUT has long been used for the treatment of headaches, gastrointestinal disorders, amenorrhea, and postpartum hemorrhage [27,28,29]. Previous studies have reported the anti-inflammatory, anti-fibrotic, anti-lipotoxic, and anticancer properties of RUT [30].

In the present study, we observed the protective effect of RUT against H_2_O_2_-induced ROS production in endothelial cells. RUT effectively regulates apoptosis by altering the balance between proapoptotic and anti-apoptotic proteins, favoring the expression of pro-apoptotic proteins. RUT enhanced the expression of Nrf2’s downstream antioxidant enzymes (HO-1, NQO1, and GCLC). Furthermore, TRPV1-PKCδ and Akt, which are critical mediators of Nrf2-dependent antioxidant response, were activated by RUT; inhibitors of these kinases (Rottlerin for PKCδ and LY294002 for Akt) attenuated Nrf2 activation. Among the PCK family, PKC-δ, PKC-α, PKC-η, and PKC-ε are particularly related to oxidative injury or cardiac protection. PKC-δ, involved in Nrf2 Ser40 phosphorylation, upregulates antioxidant enzymes [31,32]. PKCδ phosphorylation by RUT was modulated by calcium ion influx through the TRPV1 channel, suggesting that RUT likely binds to or affects the movement of calcium ions. Our previous study demonstrated that RUT induced a rapid increase in intracellular calcium concentration in endothelial cells, suggesting mobilization from internal stores such as the endoplasmic reticulum [17]. These findings, together with the current data, support the idea that RUT may modulate PCKδ activity through a combination of extracellular and intracellular dynamics. In this study, we further showed that calcium influx occurs via the TRPV1 channel, which is demonstrated to play an important role to protect the heart against apoptosis during ischemia/reperfusion through upregulating the PI3K/Akt signaling pathway [23]. For example, capsaicin, a natural TRPV1 agonist found in chili peppers, has been reported to active TRPV1-mediated signaling, contributing to cell survival and reduction of apoptosis in cardiovascular tissues [33]. Our findings suggest that RUT may influence endothelial protective signaling mechanisms not only by modulating calcium homeostasis but also through the activation of TRPV1 and downstream effectors such as PKCδ and Akt.

Moreover, AhR inhibition decreased RUT-induced Nrf2 expression and antioxidant enzyme upregulation, suggesting that RUT activates Nrf2 through an AhR-dependent mechanism parallel to the TRPV1-PKCδ-Akt axis. We established that RUT works as an AhR agonist that induces AhR translocation and CYP1A1 induction, resulting in the upregulation of Nrf2 expression and its downstream targets.

Previous studies have demonstrated the cytoprotective roles of RUT in various cellular processes. For example, in our previous study, we explored the cytoprotective effect of RUT against *t*-BHP-induced hepatotoxicity in hepatocytes and mouse livers [19]. We found that RUT inhibited ROS-mediated apoptosis in HepG2 cells by modulating the Ca^2+^-dependent signaling pathway. Specifically, RUT activates the CaMKII-PI3K/Akt pathway, which enhances the expression of antioxidant enzymes such as HO-1, NQO1, and GCL, thereby suppressing apoptosis in liver cells. The present study highlights the protective mechanism of RUT against oxidative-stress-induced apoptosis in endothelial cells via a dual-pathway regulation. The involvement of both TRPV1-mediated phosphorylation events and AhR-dependent transcriptional regulation revealed a more comprehensive defense mechanism that targets Nrf2. Nrf2 activation is a crucial mediator of the cellular defense against oxidative stress [9]. Kang et al. [34] reported that chronic intermittent hypoxia accelerates lung fibrosis in BLE-induced lung injury in an Nrf2-dependent pathway [34]. The AhR/Nrf2 pathway plays a role in protecting piglets from intestinal integrity disruption, inflammation, and oxidative stress. However, further investigation is needed to determine the in vivo effects of RUT, as well as identify the relationship between the two pathways, i.e., the TRPV1- and AhR-dependent pathways.

To summarize, our study establishes that RUT protects endothelial cells from H_2_O_2_-induced apoptosis through two distinct Nrf2-dependent mechanisms: TRPV-mediated PKCδ/Akt phosphorylation and AhR-induced Nrf2 expression. This dual pathway activation suggests that RUT is a promising candidate for mitigating oxidative stress and related vascular dysfunction.

## 5. Conclusions

In conclusion, this study established the influence of RUT on the activation of the Nrf2 signaling pathway, leading to the upregulation of antioxidant and cytoprotective genes. This activation enhances cellular defense mechanisms against oxidative-stress-induced apoptosis and suggests a potential therapeutic advantage by offering redundant protective mechanisms when using botanical extracts to control oxidative stress in vascular diseases.

## Data Availability

Data is contained within the article.

## Abbreviations

The following abbreviations are used in this manuscript:

AhR	Aryl hydrocarbon receptor
Akt	Protein kinase B
ARE	Antioxidant response element
BAPTA	1,2-Bis(o-aminophenoxy)ethane-N,N,N′,N′-tetraacetic acid
Bax	Bcl-2-associated X protein
Bcl-2	B-cell lymphoma 2
CYP1A1	Cytochrome P450 Family 1 Subfamily A Member 1
EDTA	Ethylenediaminetetraacetic acid
GCLC	Glutamate-cysteine ligase catalytic subunit
Hsp90	Heat shock protein 90
HO-1	Heme oxygenase-1
IAPs	Inhibitor of apoptosis proteins
Keap1	Kelch-like ECH associated protein 1
MAPKs	Mitogen-activated protein kinases
Nrf2	Nuclear factor-erythroid 2-related factor 2
NQO1	NADPH dehydrogenase quinone 1
PI3K	Phosphoinositide 3-kinase
PKC	Protein kinase C
ROS	Reactive oxygen species
RUT	Rutaecarpine
TRPV	Transient receptor potential cation channel subfamily V
XRE	Xenobiotic response element
